# Trans-scleral imaging of the human trabecular meshwork by two-photon microscopy

**Published:** 2011-02-24

**Authors:** David A. Ammar, Tim C. Lei, Omid Masihzadeh, Emily A. Gibson, Malik Y. Kahook

**Affiliations:** 1 Department of Ophthalmology, University of Colorado Denver, Aurora, CO; 2 Department of Electrical Engineering, University of Colorado Denver, Denver, CO; 3 Department of Bioengineering, University of Colorado Denver, Aurora, CO

## Abstract

**Purpose:**

To image the native (unfixed) human trabecular meshwork (TM) through the overlying sclera using a non-invasive, non-destructive technique.

**Methods:**

Two-photon microscopic (2PM) methods, including two-photon autofluorescence (2PAF) and second harmonic generation (SHG), were used to image through the sclera of a human cadaver eye into the TM region. Multiple images were analyzed along the tissue axis (z-axis) to generate a three-dimensional (3D) model of the region. The tissue was subsequently fixed, paraffin embedded, and histological sections were photographed for comparison to the 2PM images.

**Results:**

3D analysis of multiple 2PM SHG images revealed an open region deep within the TM consistent with the location of Schlemm’s canal (SC). Images of the scleral spur and surrounding tissues were also obtained. The SC, TM, scleral spur, and surrounding tissue images obtained with 2PM matched with histologically stained sections of the same tissue.

**Conclusions:**

2PM imaging of the outflow system of the human eye documented collagenous structures solely from inherent optical properties. 2PM successfully imaged through the sclera into the SC/TM without the need for fixation, embedding, or histological processing. This work reveals that 2PM imaging has potential as a new metric for evaluating the aqueous outflow region of the human eye and is worthy of further exploration.

## Introduction

In the conventional outflow system of the eye, aqueous humor exits the anterior chamber through the trabecular meshwork (TM), passing through Schlemm’s canal (SC) and collector channels (CC) before finally draining into aqueous veins and episcleral vessels. However, no current clinical instrumentation exists that can image the structures of this region with fine enough resolution to validate this approach as a potential metric for either diagnosing or following the progression of glaucoma. Two-photon microscopy (2PM) is a microscopy technique based on nonlinear optical processes that involves two infrared photons interacting simultaneously to excite a target molecule. Since two-photon excitation only occurs at the focus of the objective, 2PM functions as an intrinsic confocal microscope. An additional advantage of using a near-infrared laser source is deeper tissue penetration due to reduced light scattering of the longer wavelengths of light.

When 2PM is used to excite endogenous fluorophores it is called two-photon excitation autofluorescence (2PAF). 2PAF can be used to excite endogenous biologic chromophores such as NAD(P)H, collagen, elastin, and melanin [1-3]. Another nonlinear process specific to 2PM is second harmonic generation (SHG), which can only occur with non-centrosymmetric (asymmetric) macromolecules. Protein fibers such as collagen (but not elastin) can simultaneously “scatter” two lower-energy photons as a single higher energy photon. Unlike 2PAF, SHG signal occurs at a narrow blue-shifted wavelength and therefore can be separated from tissue autofluorescence using a spectral detector.

In this study we evaluate the ability of 2PM to image the TM region of human cadaver eyes. Using a long working–distance objective and optimized laser settings, we are able to penetrate the sclera and image a region of the conventional outflow system. SC and CC structures of the conventional outflow system were apparent in the three dimensional (3D) reconstructions of the two-photon images, and these structures were verified in subsequent histological sections of the same region.

## Methods

### Human eyes

Eyes from a 75-year-old male pseudophakic donor with no history of glaucoma were obtained from the San Diego Eye Bank (San Diego, CA). Approval was obtained from the Colorado Multiple Institutional Review Board for the use of human material and the tenets of the Declaration of Helsinki were followed. Informed consent was obtained from the donor or relatives for use in research.

### 2PM imaging

2PM imaging was performed using a confocal microscope (LSM 510 META on Axiovert 200M platform; Carl Zeiss MicroImaging GmbH, Göttingen, Germany) with Zeiss 510 control software (Zen; Carl Zeiss MicroImaging GmbH) equipped with a tunable mode-locked Ti:Sapphire laser (Chameleon Ultra II; Coherent Inc., Santa Clara, CA) operating at 800 nm center wavelength, with 100–200 femtosecond (fs) pulses at 80 MHz repetition rate. The excitation source (Ti:Sapphire laser) was focused on the human tissue samples by a LD “Plan-NeoFluar” 20×/0.4 NA with a working distance of 7.9 mm (Carl Zeiss MicroImaging GmbH). The emitted signal was first passed through a 650 nm short-pass filter to remove residual excitation laser light. Fluorescent signals were separated in the Zeiss META spectral detector with user-defined filter ranges of 388 nm to 409 nm for the SHG signal and 537 nm to 623 nm for autofluorescence. Image stacks were collected along the tissue axis (z-axis) and processed in the Zeiss 510 control software and proprietary software developed specifically for femtosecond imaging of the eye. Laser power used was 90% of maximum laser output (>3.5 W) and the power reaching the tissue was ~3.5 mW. This incident power and NA are within the range used by Denk et al. [4] in one of the earliest descriptions of 2PM.

### 2PM image analysis

The Zen 2009 software package (Carl Zeiss MicroImaging GmbH) was used to prepare all 2PM images and animations. This software was used to integrate the two individual signals (SHG and 2PAF) into single dual-color images as well as to adjust overall image brightness. A dual-color image stack was projected into three-dimensions (3D) using the ‘Render series’ function, creating a 3D animation of the image stack seen rotating about the y-axis. The animation and individual images from the animation were used for figures within this manuscript.

### Histology

After imaging, the section of cornea/sclera that was imaged by 2PM was excised and fixed overnight in phosphate-buffered paraformaldehyde (40 g/l paraformaldehyde, 0.2 g/l potassium phosphate monobasic, 8 g/l sodium chloride, 2.16 g/l sodium phosphate dibasic heptahydrate, pH 7.4). The tissue was embedded in paraffin, cut into ten µm-thick sections, and stained with Mayer’s hematoxylin and eosin Y (H&E: Richard-Allan Scientific, Kalamazoo, MI). Sections were photographed on a Nikon Eclipse 80i microscope (Nikon, Melville, NY) equipped with NIS-Elements software using a 10× Plan Fluor objective at 0.8× zoom with a 5 megapixel CCD (DS-Fi1; Nikon) color camera. Composite images were pieced together from overlapping photographs using commercial software (Adobe Systems Inc., San Jose, CA).

## Results

**Figure 1:**
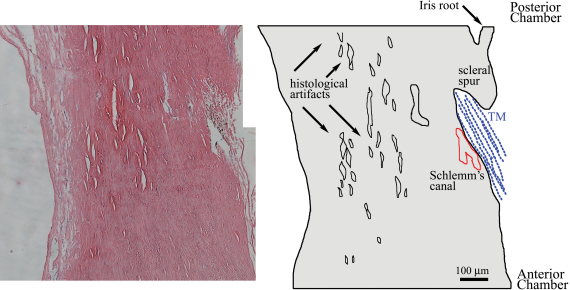
A histological section of the same human trabecular meshwork (TM) region imaged by 2PM. The paraffin section was stained with hematoxylin and eosin and placed alongside a schematic outline. The TM region is clearly visible in the lower right-hand edge of each section (toward the anterior chamber). The TM region shows darkly-stained endothelial cells populating a pink-stained fibrous tissue region of the eye. Schlemm’s canal is visible proximal to the TM region (outlined in red in schematic). The scleral spur and iris remnant are also labeled in the schematic, as are multiple opens spaces formed during processing the tissue for histology (artifacts). Black bar=100 µm.

Figure 1 shows a histological section of the sclera/TM region of a human eye stained with hematoxylin to visualize nuclei (blue-black) and with eosin Y to stain connective tissue (pink). This section was located in the region imaged by 2PM, and illustrates the characteristic collagen meshwork of the TM stained with eosin Y, and the nuclei of the cells in the TM and nearby SC. Also present are many open spaces within the scleral tissue and processing artifacts in the TM collagen fibers, which are typical findings resulting from processing the tissue for histology. The artifacts noted in Figure 1, as well as those noted by other researchers, illustrate the difficulty in relying solely on histology to examine the conventional outflow system of the human eye [5-7].

**Figure 2:**
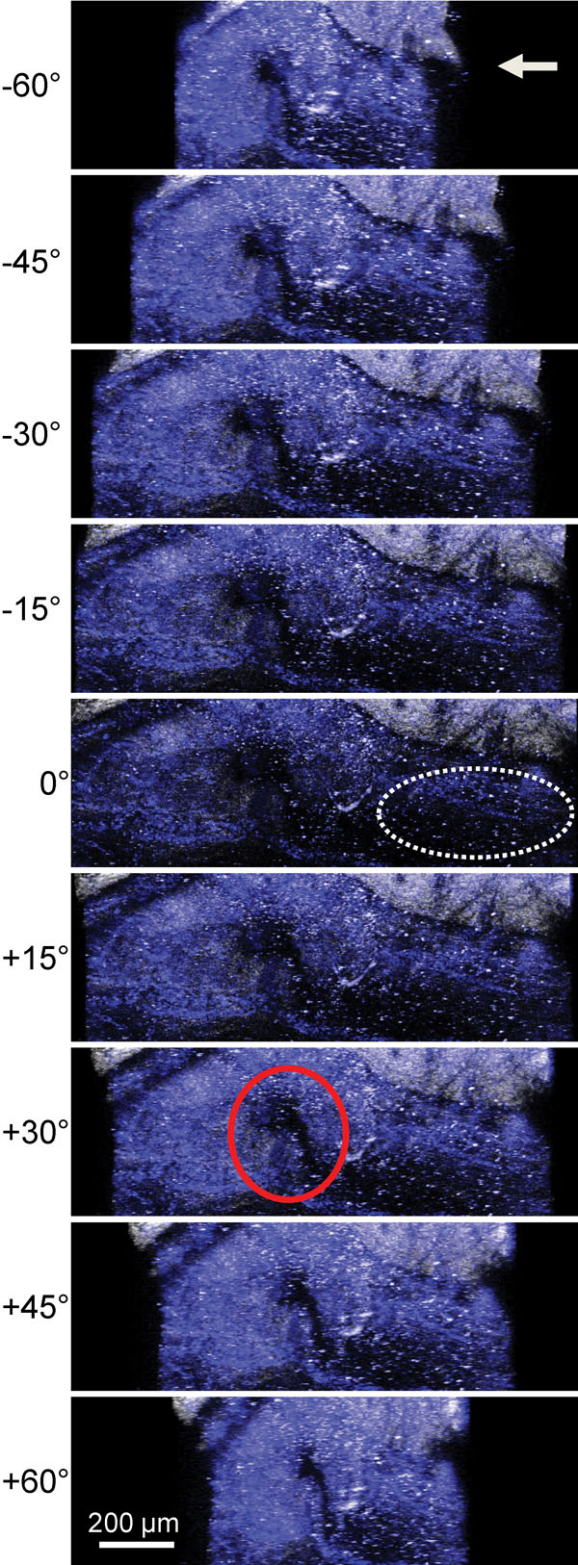
Radial sections demonstrating second-harmonic generation and autofluorescence of the TM region of a human eye. Multiple tiled scans were performed and projected into three dimensions. Shown here are snapshots every 15° of rotation about the y-axis. As the tissue rotates about the y-axis, the scleral spur, iris root (white arrow) and attached TM tissue (dotted lines) can be seen with considerable detail. Schlemm’s canal (red circle) can be seen clearly adjacent to the TM. White bar=200 µm.

**Figure 3:**
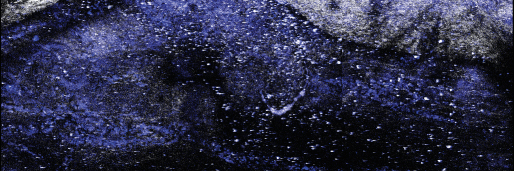
An animation representing a three-dimensional rendering of a 136.3 µm thick z-stack of the TM region of the human eye imaged through the sclera. Rotation is around the y-axis, beginning −60 °C from the original xy-imaging plane through +60 °C. Schlemm’s canal is visible in the lower center. This animation can be viewed in the html version of the article. This image is a representative frame of the animation.

2PM was performed on a human eye with the sclera facing the 20× objective. Deep tissue imaging was performed by directing the 800 nm infrared laser through the surface of the sclera at a point near the limbal region, focusing toward the TM region of the human eye. Using the META spectral detector, second harmonic generation (SHG: white) and 2-photon autofluorescence (2PAF: blue) were collected as described in the Methods to image the collagen matrix and tissue fluorescence, respectively. Each z-plane consisted of three 450×450 µm individual images stitched together (three images on the y-axis). Twenty z-planes were collected at 7.2 µm intervals for a total inclusive depth of 136.8 µm. This z-stack was computer projected into three-dimensional space and rotated 120° about the y-axis (from −60° to +60° with respect to the surface of the sclera). Radial sections of this animation were taken every 15° and shown in Figure 2. The orientation of these sections should not be confused with the radial sections generated by histology, which are tilted 90° with respect to the surface of the sclera. The scleral spur, iris root and attached TM region can be seen with considerable detail. The SC (red circle) can be seen clearly adjacent to the TM tissue (dotted lines). The complete movie is presented in Figure 3.

**Figure 4:**
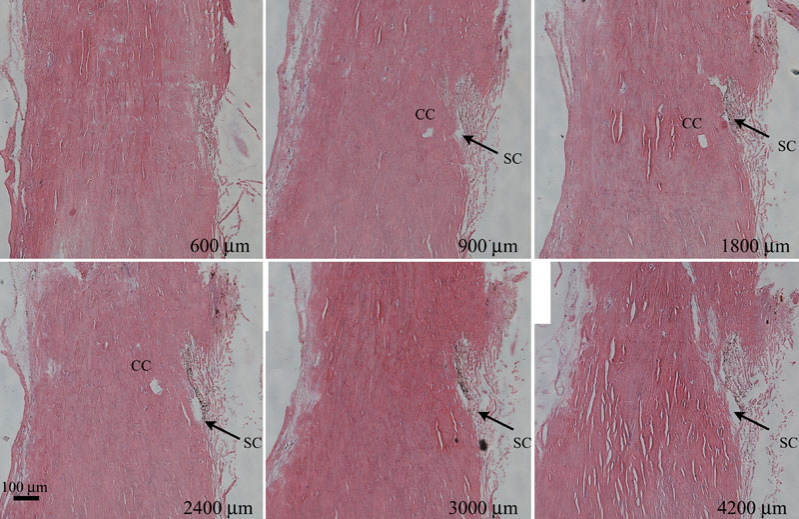
Serial sections of the TM region imaged by 2 photon microscopy. Sections shown here are indicated by the distance from the edge of the tissue block (in microns). The Schlemm’s Canal (SC) opening is apparent in many of the sections. Also prevalent is a ~50 µm round structure, representing a collector channel (CC), visible from the 900 µm section through the 2400 µm section. Black bar=100 µm.

A four by four millimeter square region of sclera/TM encompassing the region imaged by 2PM in Figure 2 was excised and prepared for histological analysis. The entire region was cut into ten micron-thick sections and stained with hematoxylin and eosin Y. Sections at regular intervals were photographed and shown in Figure 4. TM and SC are visible in each section. A CC structure is visible from the section at 900 µm through 2400 µm, running perpendicular to the SC for this distance before disappearing from view.

**Figure 5:**
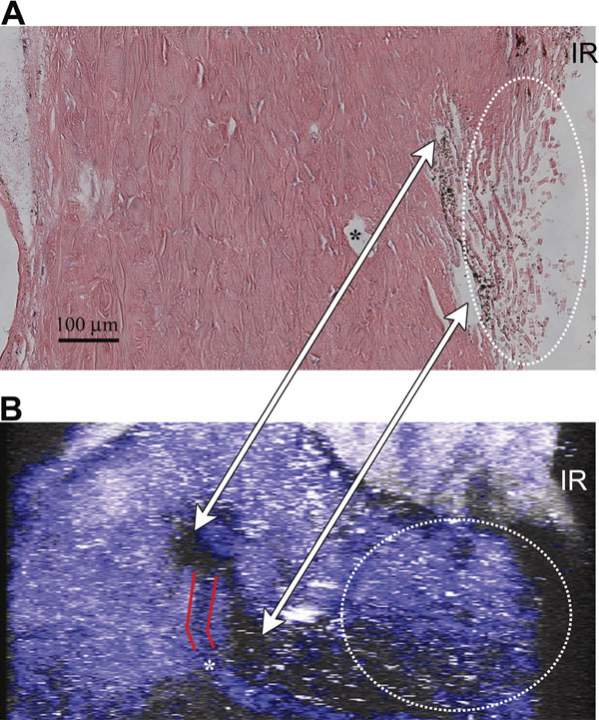
Comparison of two-photon microscopy (2PM) and conventional histology. **A**: A true radial histological section. **B**: A virtual radial-section calculated using data from the multiple z-sections imaged. The angle of this radial section is tilted 60° with respect to the face of the sclera, giving a diagonal cross section of the eye. The iris root (IR), Schlemm’s canal (SC, white arrows), collector channel (red lines and *), and TM (dotted lines) are visible in both images. The end of SC closest to the IR is visible in **B**, the 2PM image, but partially collapsed against the TM in **A**, the histological section. The connection between the collector channel and SC, outlined by red in the 2PM image in **B**, is also not visible in the histological section in **A**. Scale bar=100 µm.

Comparison of a histological stained section with the 2PM imaging is shown in Figure 5. This histological section was selected from the 450×450 µm region that was imaged by 2PM, and may not precisely represent the location of the virtual 2PM section shown in Figure 5. We found many histological landmarks in both images. For example, the SC is visible (arrow) and the TM region is indicated by dotted lines in both images. The 2PM image does not have the fine resolution showing the individual collagen fibers of the TM that is seen in the histological sample. However, the SC appears much more symmetric in 2PM, with less of the distortion seen with all histological sections shown in Figure 4 and Figure 5. Qualitatively, the channel structures of the SC appear rounder and less flattened than that seen in the histological preparations. The SC is known to be rounder near the scleral spur and narrower closer to the junction with the TM at Schwalbe’s line [8]. This shape is illustrated clearly in Figure 5 (white arrows).

We performed quantitative measurements of the structures seen in both histological sections and 2PM images. The oval-shaped structure resembling SC (Figure 5B, white arrows) spans approximately 277 µm, with a diameter of ~112 µm near the iris root, and narrowing to between ~25 µm and ~75 µm as it travels posteriorly. The SC in the histological sample has collapsed in the anterior region near the iris root (Figure 5A, upper white arrow), and is only visible posteriorly as an irregularly shaped open region with dimensions of 92 µm by 33 µm (Figure 5A, lower white arrow). We cannot ultimately conclude this represents the actual size of the SC, rather, that without the anterior collapse of the SC in the histological section this entire span of SC would be much larger and would approximate the measurements made in the 2PM images. This collapse of the anterior SC region further illustrates the artifacts that arise in histological sections. The smaller CC-like structure (Figure 5, asterisk) has dimensions of 57 µm by 33 µm in the histological section (Figure 5A) compared to 67 µm by 77 µm in the 2PM image (Figure 5B). The CC, connecting to the backside of the SC, appears fainter in the 2PM image (Figure 5B, asterisk) because it is partially obscured by intervening scleral tissue. Visible in the 2PM image, but absent from histological section, is a thin L-shaped channel connecting the SC and CC (4B, outlined by red lines). This channel is approximately 120 µm long and 33 µm in diameter.

## Discussion

In this study, 2PM was used to image the native TM region of the human eye by 2PAF and SHG through the overlying scleral tissue. We were able to image the SC as well as smaller channel-like structures that correspond to CC’s. We corroborated these physical structures in subsequent histological sections of the same tissue region. To our knowledge this is the first report of 2PM imaging of the TM region of an intact human globe eye at high enough resolution to clearly see the conventional outflow structures. Current clinical imaging of the TM region involves the use of a gonioscopic mirror, optical coherent tomography (OCT), or ultrasound biomicroscopy (UBM). Gonioscopy is limited to the aqueous surface of the TM. Commercially available OCT or UBM devices do not have the capability to image the conventional outflow pathway (SC and CC) with high enough resolution to be of clinical utility. Conventional fluorescence microscopy (single photon microscopy) uses excitation wavelengths in the visible range (400–650 nm), which undergo significant optical scattering and material absorption in biologic samples. This limits 1PM visualization to within 100 µm of the surface of the tissue (reviewed in [9]). 2PM is therefore much better suited to deep tissue imaging, and has successfully been used to image living skin to a depth of 350 µm by visualizing the 2PAF of the skin’s extracellular matrix and melanin [10].

This raises one of the greatest advantages of this technology: the ability of 2PM to image deeply into unfixed and unsectioned tissue. The resolution in this study was determined to be ~1.2 µm lateral by ~6 µm axial, which is on par with a typical 5 µm thick histological section, but has the advantage over histological sections and ultra-thin electron microscopy (EM) sections by being performed on unprocessed tissue. This eliminates potential distortions within the tissue that can occur with perfusion of fixatives, shrinkage of tissue due to alcohols, and changes to fine tissue morphology that can occur with infusion of paraffin or epoxy. These processing techniques are known to create tissue artifacts and we have noted several instances of separation of scleral tissue in our sections (Figure 1). For example, certain fixation conditions have been shown to increase the apparent number of pores found in the inner wall of SC [5], and using high-pressure freezing instead of traditional epoxy reveals sections with more extracellular matrix and less shrinkage [6]. These preparation artifacts have long been recognized as a major problem in using EM to compare healthy and glaucomatous TM specimens, so much so that in a 1968 article detailing some of the first EM images of the TM region, Spencer et al. [7] began their Discussion section posing the question, “How much of what we are seeing is artifact”? And while we cannot say a priori whether our 2PM images represent the 'true' morphology of the tissue, we can say that the tissue imaged by 2PM is in its native state.

There are multiple prior uses of 2PM within the tissues of the eye, but our work is the first to image deeply into the conventional outflow pathway. 2PM has been used to visualize retinal chromophores in the RPE and retina of the intact eye of rodents [11]. Although the long axial distance of the human eye has so far prevented in vivo imaging of the posterior chamber of the human eye, 2PAF has been performed on flat-mounts of human retina and retinal pigment epithelium (RPE) [12,13]. 2PM has been used to successfully image the human cornea [14] and Wang et al. [15,16] have performed extensive multi-photon imaging (2PAF and SHG) of the porcine cornea. In the work of the later, transient high intensity infrared laser light on the order of MW-GW/cm^2^ was focused into a 0.1 femtoliter volume (10^−15^ l), yielding high-resolution intracellular structures of the pig cornea. We estimate that with a NA of 0.4 we have a larger focus volume (~4 femtoliter) and are in MW/cm^2^ intensity range [17]; this combined with the opacity of the scleral tissue reduces the 2PM resolution compared to the images from our previous work imaging from the aqueous face of the TM [18]. Nevertheless, the authors believe that the ability to image the fluid outflow pathways deep within the sclera of an unsectioned eye represents a significant advance in ophthalmic imaging. A recent publication performed the first simultaneous 2PEF and third harmonic imaging on the cornea to detect elastin and collagen structures, and also showed the collagen and elastin structures of the TM [19]. The authors were able to discern the prominent Schwalbe’s line imaging the TM from the aqueous humor face. In contrast, we were able to perform the first deep-tissue imaging through the scleral face of the human eye.

At this time, this trans-scleral methodology is not yet suitable for clinical use because of the long time necessary to collect each z-stack of data. The long collection times also raises the question of potential tissue damage. We noted that repeated imaging of the same region of TM causes no obvious change in SHG structures on subsequent imaging. Furthermore, a large area of the imaged TM region (~4 mm) was sectioned histologically and 1) no damage was noted at the surface of the sclera or surrounding tissues and 2) the TM cells in this region appeared normal with no loss of architectural structure or evidence of thermal/coagulative damage. And finally, the wavelength of light used (800 nm) is close to the wavelength used safely by the Stratus OCT (820 nm: Carl Zeiss Mediatec AG, Jena, Germany) and other ophthalmic devices used daily in clinical practice.

The hallmark indicator of glaucoma, elevated intraocular pressure (IOP), is believed to result from dysfunction in the anterior fluid drainage system of the eye. It is clear that surrogate metrics for glaucoma, such as IOP, are not fool-proof in diagnosing disease, since 1) patients with elevated IOP often do not develop glaucoma [20] and 2) patients with glaucoma do not necessarily have high IOP [20,21]. Better metrics are needed to identify the physical state of the outflow region of the eye to identify structural abnormalities that may aid in the diagnosis of the disease, as well as monitoring the progression of drug and surgical interventions.

Multiple studies have examined the morphology of the TM in normal and glaucomatous eyes by transmission and scanning EM, with many of these studies finding differences in ultrastructure between normal and glaucomatous eyes in the outer TM, juxtacanalicular region, and inner wall of the SC [22-27]. However, in the opinion of some, these changes to the juxtacanalicular region were not large enough to account for the high aqueous flow resistance of glaucoma [28,29]. Trans-scleral imaging of this region in glaucomatous eyes may allow for further insights regarding the contribution of structures distal to the TM, such as the SC and CC, as they relate to the pathogenesis of glaucoma. 2PM is well suited to detect the electron-dense material (elastin and collagen extracellular matrix) noted in many of the above works. It may also be able to detect giant vacuoles and pores that form in the inner wall endothelium of the SC [30]. And while many of these structures have been shown to be fixative-derived artifacts in the steps required for EM [5], these structures have still been implicated in outflow resistance and likely do contribute to the disease process [31]. The ability to study the inner wall of SC in its native unfixed state, therefore, is a problem well suited for further 2PM studies and may allow investigators to definitively answer questions that remain disputed.

This study builds on our previous work that indicates that 2PM will be useful for imaging tissues responsible for the regulation, or dysregulation, of aqueous humor outflow from the eye. For the first time, 2PM has been used to image the tissue underlying the opaque sclera of a human eye. Further research is ongoing to refine the imaging technique and to optimize the collection and interpretation of data collected through proprietary software designed specifically for 2PM imaging.

## Supplementary Material

Supporting Movie
